# Appetitive behavioral traits and stimulus intensity influence maintenance of conditioned fear

**DOI:** 10.3389/fnbeh.2013.00179

**Published:** 2013-12-02

**Authors:** Megan E. Olshavsky, Carolyn E. Jones, Hongjoo J. Lee, Marie-H. Monfils

**Affiliations:** ^1^Department of Psychology, The University of Texas at AustinAustin, TX, USA; ^2^Department of Neuroscience, Center for Learning and Memory, The University of Texas at AustinAustin, TX, USA

**Keywords:** orienting, open field, fear conditioning, reconsolidation, extinction

## Abstract

Individual differences in appetitive learning have long been reported, and generally divide into two classes of responses: cue- vs. reward-directed. The influence of cue- vs. reward-directed phenotypes on aversive cue processing, is less well understood. In the current study, we first categorized rats based on their predominant cue-directed orienting responses during appetitive Pavlovian conditioning. Then, we investigated the effect of phenotype on the latency to exit a familiar dark environment and enter an unfamiliar illuminated open field. Next, we examined whether the two phenotypes responded differently to a reconsolidation updating manipulation (retrieval+extinction) after fear conditioning. We report that the rats with a cue-directed (“orienting”) phenotype differentially respond to the open field, and also to fear conditioning, depending on US-intensity. In addition, our findings suggest that, regardless of appetitive phenotype or shock intensity, extinction within the reconsolidation window prevents spontaneous recovery of fear.

## Introduction

When pairing a conditioned stimulus (CS) with a biologically significant event such as food (unconditioned stimulus, US), rats develop conditioned responses (CR). In the case of light-food pairings, some rats develop both CS- and US-directed responses, that is, they orient/rear toward the light cue and approach the site of food delivery, while other rats develop only the food cup approach behavior. Because both groups exhibit an approach to the food cup and only a subset develops an orienting response to the light, we characterize these groups based on their conditioned orienting response to the CS and classify them as Non-orienters and Orienters, respectively.

Numerous reports, including our own, have indicated that these two phenotypes differ in measures of risky decision making, delay discounting, novelty preference, dopaminergic response to cues, and response to drug exposure (Flagel et al., 2011; Lovic et al., 2011; Olshavsky et al., 2012; Schiller et al., 2012). Orienters and Non-orienters also behave differently in their susceptibility to appetitive memory updating (Olshavsky et al., 2013). Monfils et al. (2009) previously showed that presenting an isolated retrieval trial (CS) prior to an extinction session led to a persistent reduction in fear expression, which did not leave the fear memory susceptible to spontaneous recovery (SR), reinstatement, or renewal. Unlike standard extinction, the retrieval+extinction procedure has been proposed to involve an updating of a memory during the reconsolidation window (Monfils et al., 2009; Schiller et al., 2010). The isolated retrieval trial is thought to induce memory destabilization for a limited time period during which the memory is labile (Monfils et al., 2009; Nader et al., 2000). Using a procedure based on this paradigm (Monfils et al., 2009; Schiller et al., 2010), Olshavsky et al. (2013) observed that rats receiving a retrieval trial prior to extinction showed attenuated conditioned responding during tests for SR (Olshavsky et al., 2013). Interestingly, this effect was dependent on whether the rats were Orienters or Non-orienters—only Orienters showed attenuation of conditioned responding after the retrieval-extinction procedure. This result is particularly important in light of the fact that many (Clem and Huganir, 2010; Schiller et al., 2010; Rao-Ruiz et al., 2011) but not all (Chan et al., 2010) labs have observed the persistent fear memory updating described in Monfils et al. (2009), prompting a need to investigate the boundary conditions that surround this form of memory updating. To this effect, for the present study we first classified rats as either Orienters or Non-orienters based upon their expression of either CS-directed or US-directed responses during light-food pairings, we then compared their behaviors within an open field task, then tested whether expression of conditioned fear differs in rats that show robust cue-oriented responding and those that do not, and finally, examined whether fear memory could be persistently attenuated in those groups using the retrieval+extinction paradigm (Monfils et al., 2009).

## Materials and methods

### Subjects

Sixty-six Long-Evans male rats (250–275 g upon arrival, Charles River Laboratories) were used. Rats were maintained on a 12-h regular light-dark cycle with lights on at 7am. For the open field and appetitive conditioning portions of the experiment, rats were maintained at 90% free-feeding weight; water was available *ad libitum*. During fear conditioning procedures, food and water were both provided *ad libitum*. All experiments were conducted according to *the National Institutes of Health's Guide for the Care and Use of Laboratory Animals*, and the protocols were approved by the Institutional Animal Care and Use Committee at the University of Texas at Austin.

Initially, rats were trained to retrieve food pellets from a food cup located within an appetitive conditioning chamber. Eight individual conditioning chambers (30.5 W × 25.4 D × 30.5 H in cm, Coulbourn Instruments, Allentown, PA) with aluminum sidewalls and ceiling, clear acrylic front and back walls and stainless steel rod floors (rods 0.5 cm in diameter, spaced 1.0 cm apart) comprised the appetitive conditioning context. A wall-mounted magazine delivered grain pellets (Test Diet, 45 mg) to a recessed food cup mounted 2.5 cm above the floor. Each chamber was enclosed in a light- and sound-attenuated box (58.4 × 61 × 45.7 cm); a ventilation fan provided masking noise. A video camera was mounted within each box and images were recorded during behavioral training. During the initial food cup training a total of 30 pellets were delivered to the food cup at a variable intertrial interval (ITI) averaging 60 s over a 30-min session. After one session, all rats reliably retrieved the grain pellets.

### Open field

**Figure 1:**
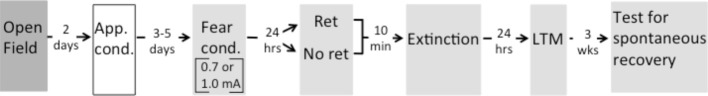
**Timeline of experimental design**. Rats were first tested for their willingness to enter an illuminated open field. Rats then received appetitive conditioning (App. cond.) with 56 light-food pairings in Context A. On their last day of appetitive conditioning rats were classified as Orienters and Nonorienters. After 3–5 days, both groups were fear conditioned (Fear cond.) with 3 tone-shock pairings of either 0.7 or 1.0 mA in Context B (indicated by gray shading). 24 h after fear conditioning, rats were exposed to a single cue retrieval trial (Ret) or a typical extinction session (No ret). For rats in the Ret group that received a cue exposure and those in the No ret group that received a context exposure, the exposure occurred 10 min prior to beginning the extinction session. 24 h after extinction, rats were tested for long-term memory (LTM), and 3 weeks later tested for spontaneous recovery. Context change is indicated by shading.

After food cup training, both rats' latency to enter an illuminated open field and their preference for the illuminated open field vs. a familiar dark compartment were assessed. Two open field chambers consisting of white acrylic floors surrounded on all sides by clear acrylic walls were used (43.2 W × 43.2 D × 30.5 H in cm). On day 1, rats were restricted to an opaque black insert (43.3 W × 21.6 D × 30.5 H in cm) for 10 min. The following day rats were initially placed within the black insert, but were free to exit into the illuminated portion of the open field and had 10 min of free access to both sides. Activity in both sides of the field was detected by infrared beam motion detectors (Figure 1).

### Appetitive conditioning

Forty-eight hours after completing the open field test, rats began appetitive conditioning. The first day of appetitive conditioning consisted of two parts. In order to habituate the unconditioned orienting response to light, the stimulus light (2-Watt white light mounted 20 cm above the magazine) was illuminated eight times, for 10 s each time, without any food pellets being delivered to the magazine. Then, during the second half of the session, 10 s light-CS illuminations were followed by grain pellet delivery into the food cup. For the next three days of conditioning, sessions consisted of 16 light–food pairings with a variable ITI averaging 120 ± 50 s.

Nosepoke to the food cup was detected by an infrared beam at the opening, while orienting behavior was scored by a blind observer from DVD recordings of sessions. Orienting measures were directly adapted from the ones used by Holland and colleagues (Gallagher et al., 1990; Lee et al., 2005, 2010, 2011). Even though the light-CS was a localized cue, it still provided diffuse illumination of the entire chamber. Thus, an orienting response was defined as any rearing response in which both forepaws were lifted from the floor of the training box, but did not include grooming behavior. For each light-food trial, behavior was sampled at every 1.25 s resulting in 12 observations: 4 times during the 5 seconds immediately preceding the onset of the CS (preCS), 4 times during the first 5 s of the CS (CS1), and 4 times during the last 5 s of the CS (CS2). Because orienting response and food cup approach occur predominantly during CS1 and CS2, respectively (Holland, 1977), we report orienting response from CS1 and food cup approach behavior from CS2. Their behaviors during preCS are subtracted to account for any baseline differences (Figure 1).

### Fear conditioning

Following appetitive training, rats were transferred to a new colony and after a 3–5 days of acclimation, all rats were fear conditioned in a second context. All remaining procedures (fear conditioning, long-term memory test, and the test for SR) were conducted in this second context. Rats were fear conditioned in chambers equipped with two metal walls, two clear plexi-glass walls, and stainless-steel rod floors connected to a shock generator (Coulbourn Instruments, Allentown, PA). Each conditioning chamber was enclosed in an acoustic isolation box (Coulbourn Instruments) and lit with a red house light. Behavior was recorded with digital cameras mounted on the top of each unit. Stimulus delivery was controlled using Freeze Frame software (Coulbourn Instruments). The CS used for fear conditioning was a 20-s tone (5 kHz, 80 dB). The US was either a 0.7 or 1.0 mA footshock 500 ms in duration. Orienters and Non-orienters, as determined by the orienting response during the last eight trials of appetitive training, were divided into two shock intensity groups for fear conditioning (0.7 and 1.0 mA). On the fear-conditioning day, after a 2-min habituation period, all rats received three 20-s presentations of the tone CS (variable ITI = 120 s), each co-terminating with either a 0.7 or 1.0 mA foot-shock. An experimenter blind to group assignment scored freezing behavior manually from video recorded during each session. Freezing was defined as the absence of any movements, excluding those required for respiration. The total number of seconds spent freezing throughout the CS presentation was expressed as a percentage of CS duration.

Twenty-four hours after fear conditioning, all subjects underwent either extinction (ext only) or retrieval+extinction (ret+ext). For the extinction session, rats were placed in the fear-conditioned context and exposed to 19 non-reinforced presentations of the tone CS (variable ITI = 120 s). A subset of these rats (*n* = 21 out of 37) in the extinction only group were placed in the context 10 min prior to the extinction session but received no CS presentations. Context-exposed and non-context-exposed rats from the No Retrieval groups were not significantly different and these groups were collapsed for the remainder of analyses. Rats in the ret+ext group were first exposed to a single CS presentation in the fear-conditioned context, returned to the home-cage for 10 min, and then returned to the same context for the remaining 18 extinction trials. This resulted in eight groups for analysis - Orienter 0.7 mA ret+ext *n* = 8; Orienter 0.7 mA ext only *n* = 9; Non-orienter 0.7 mA ret+ext *n* = 9; Non-orienter 0.7 mA ext only = 7; Orienter 1.0 mA ret+ext *n* = 8; Orienter 1.0 mA ext only *n* = 9; Non-orienter 1.0 mA ret+ext *n* = 9; Non-orienter 1.0 mA ext only *n* = 8 (Figure 1).

## Results

### Appetitive conditioning

**Figure 2:**
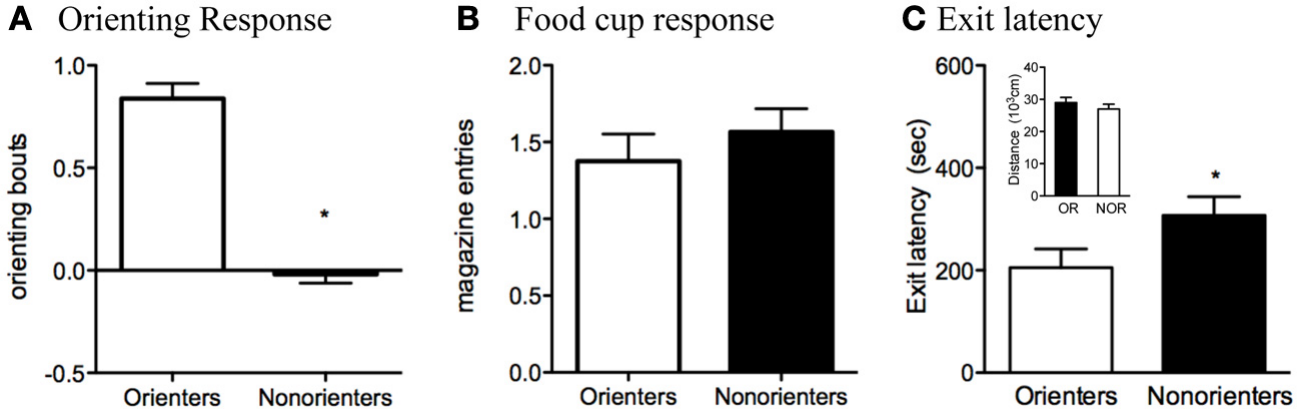
**(A,B)** Conditioned orienting and food cup approach for the Orienters and Nonorienters. Mean ± s.e.m number of orienting bouts (^*^p < 0.0001) **(A)** or food cup entries (*p* = 0.42) **(B)** averaged for last 8 trials of training. Orienters showed significantly more orienting than Nonorienters, but the food cup response was equivalent between groups. **(C)** Latency to exit the dark insert and enter the illuminated open field. Orienters exited significantly more quickly than the Nonorienters (*p* = 0.05). Activity, as measured by the total distance traveled within both fields **(C)**, did not differ between Orienters and Nonorienters (*p* = 0.37).

Based on their average number of orienting bouts during the last eight trials of training, rats were divided into two groups. Rats scoring at or above the median (0.38 bouts/trial) were classified as Orienters (*n* = 34), while those rats that scored below the median were classified as Non-orienters (*n* = 32). The mean conditioned orienting levels, 0.85 ± 0.07 and −0.01 ± 0.04, were significantly different between Orienters and Non-orienteres, respectively, *t*_(64)_ =9.84, *p* < 0.0001 (Figure 2A). Groups of rats, however, did not differ in displaying conditioned food cup approach (Figure 2B). Furthermore, the groups did not differ in unconditioned orienting response during the first 8 trials, in which light was presented without any food: Mean orienting bouts during those trials were 0.36 for Orienters and 0.35 for Non-orienters (*p* = 0.91) (data not shown).

As stated in the materials and methods section (2.3. Appetitive conditioning), these reported numbers reflect elevated scores, in which the behaviors in the absence of CS were subtracted from the ones during CS presentation. Analyses of preCS responses (i.e., orienting and food-cup behavior during the 5-s immediately before the CS onset) revealed no differences between Orienters and Non-orienters (*p* > 0.05). PreCS orienting bouts were 0.24 (Orienters) and 0.35 (Non-orienters) and preCS food-cup numbers were 0.46 (Orienters) and 0.49 (Non-orienters). Furthermore, orienting scores during the first half (CS1) and food-cup scores from the second half (CS2) of CS presentation are presented in Figure 2, due to the predominant display of these behaviors in respective time points. Further analyses of these two behaviors in both CS1 and CS2 with repeated ANOVA of two CS time points still revealed the same trend in which there was an overall significant difference in orienting response between Orienters and Non-orienters, *F*_(1, 64)_ = 54.4, *p* < 0.001, but not in food-cup behavior, *F*_(1, 64)_ = 3.47, *p* > 0.05. As expected, the overall orienting levels were significantly higher during CS1 compared to CS2, *F*_(1, 64)_ = 8.60, *p* < 0.01, and the food-cup response was significantly higher during CS2 compared to CS1, *F*_(1, 64)_ = 42.4, *p* < 0.001.

### Open field

Analysis of data collected during the dark-light open field task indicated that Orienters exited the dark insert (and entered the illuminated field) more quickly than Non-orienters, *t*_(64)_ = 1.98, *p* = 0.05 (Figure 2C). There was also a trend for Orienters to spend more time in the illuminated field than Non-orienters, *t*_(64)_ = 1.85, *p* = 0.07. These results cannot be attributed to a difference in general activity levels, as the ambulatory distance traveled of the two groups were comparable, *t*_(64)_ = 0.91, *p* = 0.37 (Figure 2C).

### Fear conditioning

**Figure 3:**
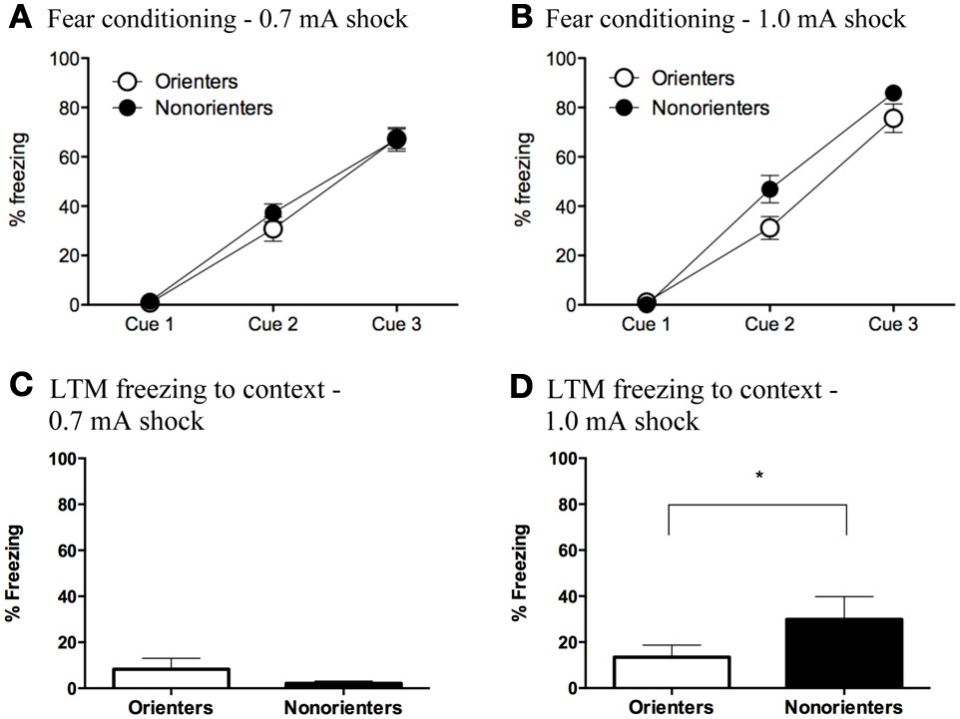
**(A,B)** Freezing during fear conditioning with a 0.7 mA and 1.0 mA footshock. **(A)** Orienters and Non-orienters showed no differences in freezing during conditioning when the US was a 0.7 mA footshock (*p* = 0.49). **(B)** Non-orienters froze significantly more than Orienters during fear conditioning when the US was 1.0mA footshock (*p* = 0.04). Each conditioning session involved three CS-US pairings. (**C,D**) Contextual freezing 24 h after fear conditioning to either a 0.7 or 1.0 mA footshock. **(C)** There were no significant differences between Orienters and Non-orienters in freezing to the fear conditioning context when the US was 0.7 mA footshock (*p* = 0.09) and overall context freezing was extremely low. **(D)** Non-orienters froze significantly more than Orienters to the fear conditioning context when the US was a 1.0 mA footshock (^*^p = 0.03).

Freezing during the fear conditioning session was analyzed using mixed factor ANOVAs with fear conditioning cue (3 cues total) as the repeated measure and orienting classification (Orienter or Non-orienters) and shock intensity (0.7 or 1.0 mA) as the between subjects factors. There was a significant within-subjects effect of fear conditioning cue, *F*_(2, 116)_ = 391.58, *p* < 0.001, indicating that rats froze significantly more toward the end of the fear conditioning session than at the beginning. Additionally, overall rats froze significantly more throughout conditioning to the 1.0 mA than the 0.7 mA. In addition the Orienters and Non-orienters were differentially affected by shock intensity. There was a significant fear conditioning cue x shock intensity interaction, *F*_(2, 116)_ = 3.74, *p* = 0.027 as well as an overall main effect of both orienting classification, *F*_(1, 58)_ = 4.17, *p* = 0.046, and shock intensity, *F*_(1, 58)_ = 5.36, *p* = 0.024. Follow up ANOVAs for each shock intensity revealed that for the 0.7mA fear conditioning group (Figure 3A), there were no differences in freezing levels during acquisition between the Orienters (*n* = 15) and Non-orienters (*n* = 14), *F*_(1, 27)_ = 0.49, *p* = 0.49. However, rats classified as Orienters who were fear conditioned to the 1.0 mA shock (*n* = 17) froze significantly less than rats classified as Non-orienters (*n* = 16) evidenced by an overall main effect of orienting on freezing levels during the fear conditioning session, *F*_(1, 31)_ = 4.57, *p* = 0.041 (Figure 3B). However, a comparison of the mean freezing of Orienters and Non-orienters in the 1.0 mA group revealed that the groups were not significantly different during the last trial of conditioning.

### Contextual fear

Contextual fear was measured by scoring freezing during a 20 s sample within the first 2 min that the rat was placed in the fear conditioning context the day after fear conditioning. For rats that received a CS retrieval, freezing to the context was measured in the 20 s immediately preceding the CS onset. In the ext only group, rats that received a context exposure only, freezing to the context was measured for 20 s at the same time point as the retrieval group. In the subset of animals that did not receive a context exposure, context freezing was measured in the 20 s preceding the first CS of extinction. All of these measurements took place at the same time point during the rat's first exposure to the fear conditioning context. A 2 × 2 ANOVA with orienting classification and shock intensity as the factors revealed a significant main effect of shock intensity, *F*_(1, 62)_ = 15.96; *p* < 0.001, no main effect of orienting classification, *F*_(1, 62)_ = 1.90; *p* = 0.173, and an orienting classification X shock intensity interaction, *F*_(1, 62)_ = 7.73; *p* = 0.007. Follow up *t*-tests revealed that there were no significant differences between Orienters and Non-orienters after conditioning to a 0.7mA shock, *t*_(31)_ = 1.74; *p* = 0.092, and overall contextual freezing levels were very low (<10%) as seen in Figure 3C. However, after conditioning to a 1.0 mA footshock, Non-orienters showed significantly more freezing to the context than Orienters, t_(31)_ = 2.27; *p* = 0.03 (Figure 3D).

### Extinction/retrieval+extinction

**Figure 4:**
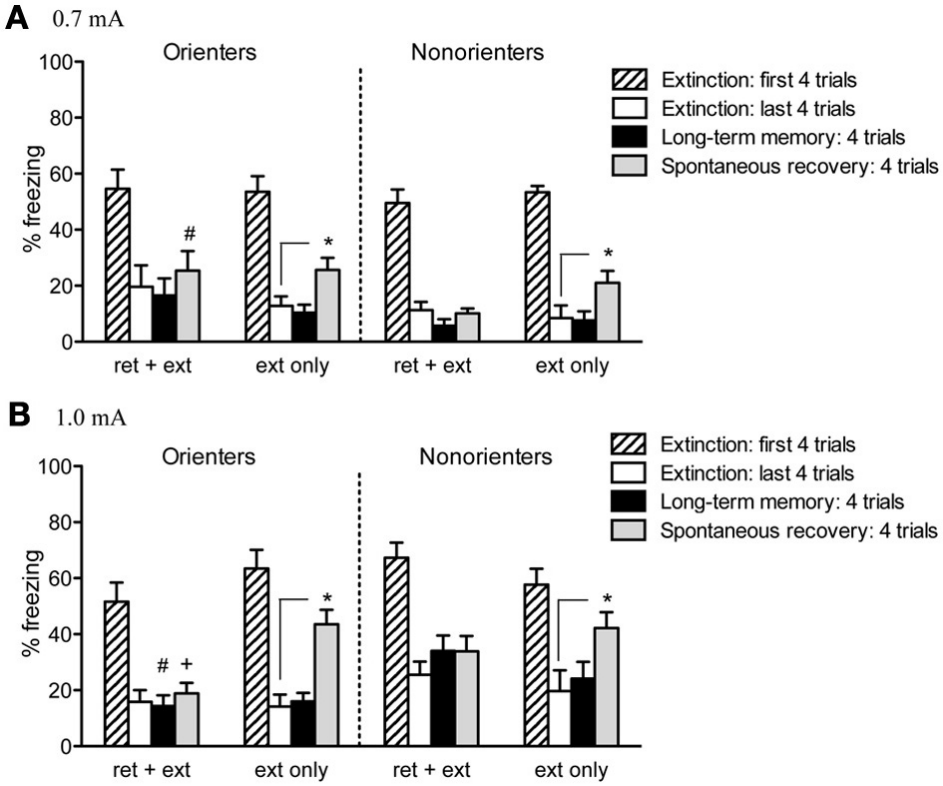
**Cue-induced freezing at the beginning of extinction, end of extinction, during LTM test, and spontaneous recovery. (A)** For rats conditioned with a 0.7 mA shock, a retrieval trial prevented spontaneous recovery (i.e., there was no significant increase in freezing from the end of extinction to spontaneous recovery test; Orienters *p* = 0.206, Non-orienters *p* = 0.732). While neither group showed significant spontaneous recovery, Non-orienters froze significantly less than Orienters during the test for spontaneous recovery (^#^*p* = 0.041). Rats receiving typical extinction treatment did show a significant increase in freezing (Orienters *p* = 0.014, Non-orienters *p* = 0.032). **(B)** Rats conditioned with a 1.0 mA shock showed the same pattern of results: a retrieval trial prior to extinction attenuated spontaneous recovery (Orienters *p* = 0.524, Nonorienters *p* = 0.235). Rats exposed to typical extinction showed a significant increase in freezing (Orienters *p* < 0.001, Non-orienters *p* = 0.032). While neither Orienters nor Non-orienters that received ret+ext showed significant increases in freezing from the end extinction to LTM or spontaneous recovery tests, Orienters showed significantly less freezing than Non-orienters at both time points (LTM ^#^*p* = 0.011, spontaneous recovery ^+^*p* = 0.045).

Given the differences between Orienters and Non-orienters in freezing during the 1.0 mA fear conditioning session, we compared the mean of the first four trials of extinction and tested whether our groups differed in their fear conditioning retention. Neither orienting classification, shock level, nor retrieval group resulted in any significant differences in freezing during the first 4 trials of extinction (*ps* > 0.05) suggesting that the differences observed during fear acquisition are a result of differential responses to the immediate presence of the foot-shock as opposed to differences in the ability to acquire and retain CS-US association. Freezing during the extinction session was initially analyzed with a 2 × 2 × 2 mixed factor ANOVA with extinction cue as the repeated measure and retrieval group (ext only, ret+ext), orienting classification (orienters or non-orienters), and shock intensity (0.7 or 1.0 mA) as the between subjects factors. Rats did show a significant reduction in freezing over the course of extinction as evidenced by a significant within-subjects effect of extinction cue, *F*_(18, 1026)_ = 62.53, *p* < 0.001, with no overall main effect of either orienting classification, *F*_(1, 57)_ = 0.05, *p* = 0.831, or retrieval group, *F*_(1, 57)_ = 2.40, *p* = 0.127 (Figure 4).

### Long term memory of fear

Twenty-four hours after extinction, rats were tested for long-term memory (LTM) by presenting 4 tone-only trials (variable ITI = 120 s) in the same context as fear conditioning and extinction. Freezing behavior during these trials was scored and averaged During the LTM test, none of the experimental groups showed a significant increase in freezing, as compared to their own freezing at the end of extinction (all *p*'s > 0.1). For rats conditioned with a 0.7 mA shock, no between-group differences existed in LTM freezing. For rats conditioned with a 1.0 mA shock, the freezing levels of Orienters and Non-orienters receiving typical extinction treatment (ext only) were comparable; however, Non-orienters in the ret+ext group showed significantly higher freezing than Orienters in the ret+ext group, *t*_(15)_ = 2.89, *p* = 0.011 (Figure 4).

### Spontaneous recovery of fear

Twenty-one days after extinction, rats were returned to the chambers and tested for SR of freezing by playing 4 tone-only trials (variable ITI = 120 s). An overall ANOVA with orienting classification, shock intensity, and retrieval group as the factors revealed no overall effect of orienting classification, *F*_(1, 57)_ = 0.19; *p* = 0.661, but did reveal a significant overall effect of both retrieval group, *F*_(1, 57)_ = 10.02; *p* = 0.002, and shock intensity, *F*_(1, 57)_ = 16.05; *p* < 0.001, as well as a significant orienting classification X shock intensity interaction, *F*_(1, 57)_ = 5.75; *p* = 0.02, and a trend toward an orienting classification X shock intensity X retrieval group interaction, *F*_(1, 57)_ = 3.73; *p* = 0.058 (Figure 4). Rats receiving typical extinction treatment (ext only) showed recovery of freezing, regardless of orienting classification or shock intensity, i.e., freezing was significantly increased from extinction to the SR test [Orienters—0.7 mA: *t*_(8)_ = 3.133, *p* = 0.014; Non-orienters—0.7 mA: *t*_(5)_ = 2.96, *p* = 0.032; Orienters—1.0 mA: *t*_(8)_ = 7.73, *p* < 0.001; Non-orienters—1.0 mA: *t*_(6)_ = 2.785, *p* = 0.032]. In contrast, rats exposed to a retrieval trial prior to extinction did not show significant recovery of freezing during the SR test regardless of orienting classification or shock intensity (all *p's > 0.2*). Although neither Orienters nor Non-orienters receiving a retrieval trial prior to extinction showed a significant increase in freezing from extinction to SR test, for either shock intensity, Non-orienters showed more freezing behavior during SR test than Orienters after conditioning to a 1.0mA shock, *t*_(15)_ = 2.18, *p* = 0.045, and less freezing behavior than Orienters after conditioning to a 0.7mA shock, *t*_(15)_ = 2.23, *p* = 0.041.

## Discussion

Fear conditioning provides a controlled means to investigate aversive associations that underlie many pathological fear conditions. Memory update methods such as ret+ext, where an extinction session is presented within the reconsolidation window show promise for reducing fear non-invasively; however, individual differences between subjects and methodological variations across laboratories leaves the efficacy of such paradigms in question. Here we consider how individual differences in response style during an appetitive conditioning task (i.e., propensity for conditioned orienting to a light stimulus predictive of food) relate to individuals' hesitance to enter an open field and how they affect freezing after fear conditioning. We report that Non-orienters show more reluctance to enter an illuminated open field, indicating an enhanced fear of unfamiliar open environments, as compared to Orienters. Additionally, we report that when conditioned with a tone and 1.0 mA footshock, Non-orienters show heightened freezing. Groups do not differ in their response conditioning with a 0.7 mA shock.

After fear conditioning to a foot-shock of either standard intensity (0.7 mA) or increased intensity (1.0 mA), ret+ext prevented SR of freezing for both Orienters and Non-orienters. However, for the 1.0 mA experiment, Non-orienters in the ret+ext group froze significantly more than Orienters in the ret+ext group. We show that while retrieval+extinction prevents the significant return of fear for both phenotypes, the intensity of the US used in training and subjects' appetitive phenotype affect the magnitude of fear behavior that persists. A relationship between these two behaviors (conditioned orienting in an appetitive task and fear expression in a fear conditioning task) seems perhaps unsurprising given the overlap in the neural circuitry responsible for each. Projections from the central nucleus of the amygdala have been shown to be necessary for both the acquisition of conditioned orienting to a cue predictive of reward and the freezing response exhibited after fear conditioning (Ledoux et al., 1988; Gallagher et al., 1990; Han et al., 1997; Goosens and Maren, 2001; Choi and Brown, 2003; Duvarci et al., 2011). It is possible Orienters and Non-orienters have fundamental differences in central amygdala function and that the results reported here are evidence of that variation, but more investigation needs to be done.

Furthermore, we report that after conditioning to a strong 1.0 mA footshock, Non-orienters show increased susceptibility to condition to context than Orienters as evidenced by increased freezing in the absence of the CS when returned to the chamber 24 h after conditioning. This result replicates previous research indicating that goal-trackers show more context-induced freezing when placed in the conditioning context 24 h after aversive conditioning (Morrow et al., 2011). However, the same study also reported that sign-trackers show more cue-induced freezing when first re-exposed to an aversive CS, while we report that the two groups show no difference when initially re-exposed to the tone. Morrow et al. (2011) reports freezing results during re-exposure to the CS in a novel context, 24 h after conditioning, while we report freezing during CS exposure in the original conditioning context both 24 h after conditioning and 21 days after extinction or retrieval+extinction. Another difference lies in characterization of sign-tracking phenotypes. Morrow et al., used insertion of an inactive lever as a CS which elicited a different form of sign-tracking behavior (i.e., engagement with the lever). Unlike Orienters that also displayed US-directed food-cup behavior, these rats engaged almost exclusively with the lever while others engaged almost exclusively with the food cup resulting in an inverse correlation between these two behaviors. These two types of sign-tracking behaviors (i.e., lever-engagement and orienting) might represent slightly different phenotypes. It has been shown that the central nucleus of the amygdala, which is crucial for acquisition of conditioned orienting (Gallagher et al., 1990), is not necessary for sign-tracking behavior toward the lever CS (Chang et al., 2012).

Non-orienters' apprehension about entering an open field, enhanced freezing during fear conditioning, and enhanced expression of contextual fear suggest that their expression of fearful behaviors differs from that of Orienters across modalities and circumstances. Although retrieval+extinction prevents SR in all cases, conditioning to a 1.0 mA footshock resulted in Non-orienters freezing more than Orienters during tests both 24 h (LTM) and 21 days (SR) after retrieval+extinction, whereas conditioning to a 0.7 mA foot shock resulted in Orienters freezing more than Non-orienters during a test 21 days after retrieval+extinction. These differences in freezing after conditioning to a 0.7 mA foot shock were not present 24 h after retrieval+extinction. Combined, our results suggest that time, orienting phenotype, and shock intensity all interact to influence the ability of an extinction session within the reconsolidation window to update an existing fear memory trace. The influence of these factors on the efficacy of retrieval+extinction may provide some explanation for the variation in reported results for fear memory updating studies. Despite the fact that, when systematically measured there is no significant effect of orienting phenotype on the efficacy of the retrieval+extinction paradigm to prevent the return of fear, it is plausible that, in the absence of explicitly observing and quantifying orienting phenotypes, these factors might still contribute to group differences. Orienting-driven effects could occur, for instance, in a case where we have an unintended uneven (and unnoticed) distribution of Orienters/Non-orienters across experimental groups. Interestingly, the orienting phenotype seems to differentially affect fear vs. appetitive memory updating. It would be important, going forward, to examine other potential factors that might contribute variability in orienting phenotype (e.g., rat strain). Ultimately, we believe that understanding individual differences and their neurobiological correlates is key to optimizing memory update techniques.

### Conflict of interest statement

The authors declare that the research was conducted in the absence of any commercial or financial relationships that could be construed as a potential conflict of interest.
